# Methylation of SPRED1: A New Target in Acute Myeloid Leukemia

**DOI:** 10.3389/fonc.2022.854192

**Published:** 2022-03-10

**Authors:** Nan Su, Yujiao Wang, Xianglan Lu, Weihong Xu, He Wang, Wenbin Mo, Hui Pang, Rurong Tang, Shibo Li, Xiaojing Yan, Yan Li, Rui Zhang

**Affiliations:** ^1^ Department of Hematology, The First Affiliated Hospital of China Medical University, Shenyang, China; ^2^ Department of Pediatrics, University of Oklahoma Health Sciences Center, Oklahoma City, OK, United States; ^3^ Department of Anesthesiology, The First Affiliated Hospital of China Medical University, Shenyang, China

**Keywords:** SPRED1, AML, THP-1, hypermethylation, 5-AZA

## Abstract

Sprouty-related, EVH1 domain-containing protein 1 (SPRED1) has been identified as a novel tumor suppressor gene in acute myeloid leukemia (AML). Previous studies showed that SPRED1 methylation levels were significantly increased in AML patients, making it an interesting candidate for further investigations. To confirm the association of SPRED1 methylation, clinical parameters, and known molecular prognosticators and to identify the impact of methylation level on treatment outcome, we conducted this study in a larger cohort of 75 AML patients. Significantly increased methylation levels of SPRED1 were detected at four of ten CpG units by quantitative high-resolution mass spectrometry-based approach (MassARRAY) in AML patients. Whereas overall survival (OS) and relapse-free survival (RFS) showed no statistical difference between hypermethylation and hypomethylation subgroups, the relationship between methylation level and treatment response was indicated in paired samples from pre- and post-induction. To determine the possible mechanism of SPRED1 methylation in AML, we performed *in vitro* experiments using THP-1 cells, as the latter showed the highest methylation level (determined by utilizing bisulfite modification) among the three AML cell lines we tested. When treated with 5-AZA and lentivirus transfection, upregulated SPRED1 expression, decreased cell proliferation, increased cell differentiation and apoptosis, and inactivated phosphorylated extracellular signal-regulated kinase (p-ERK) were detected in THP-1 cells. These results show that demethylation of SPRED1 can inhibit the proliferation of AML cells and promote their differentiation and apoptosis, possibly by the ERK pathway. The hypermethylation of SPRED1 is a potential therapeutic target for AML.

## Highlights

- Hypermethylation of SPRED1 is widely observed in AML and associated with treatment responses.
- Demethylation of SPRED1 can reduce proliferation and promote differentiation and apoptosis of AML cells.

## Introduction

Epigenetic modifications, such as abnormal DNA methylation, are believed to play an important role in the occurrence and development of acute myeloid leukemia (AML) (1–3). A majority of studies have shown that the abnormal DNA methylations of tumor suppressor genes (TSGs) and proto-oncogenes serve as a prognostic marker of AML (4, 5). Especially hypermethylation could also serve as a therapeutic target, which frequently occurs in some subtypes of AML, such as in the elderly or in those with myelodysplastic-related or AML with t(8;21) (6, 7). As an alternative or a complementary choice with traditional chemotherapy, demethylating agents supply an opportunity for longer survival in some specific types of AML (8, 9). However, there are still unknown factors for demethylating therapy, such as the duration of treatment, the markers of demethylating response, and the poor outcomes of treatment (10). One of the main reasons is that little is known about the targets of demethylating agents themselves, and the specific genes that are hypermethylated drive the leukemogenesis.

The Sprouty-related, EVH1 domain-containing protein 1 (SPRED1) encoded by this gene is a member of the Sprouty protein family and is phosphorylated by tyrosine kinase in response to several growth factors. Also, it is able to inhibit the Ras/Raf/ERK pathway as a negative growth factor and is regulated by cytokine and chemokine-induced ERK (11). In a previous study, we have proved the adverse impact of downregulation of SPRED1 on AML (12). Although loss-of-function frameshift mutation of SPRED1 was first reported in a child AML with Legius syndrome (13), neither mutation nor deletion of SPRED1 was common in AML (12). Recently, we found that the SPRED1 promoter regions were hypermethylated in adult AMLs and were negatively correlated with the expression level of SPRED1 mRNA (14). However, further evidence on abnormal methylation of SPRED1 from a large cohort of patients is needed to clarify its clinical and prognostic significance.

In this study, we expanded our clinical samples, collected 75 *de novo* AML [non-acute promyelocytic leukemia (non-APL)], and detected the methylation degree of SPRED1 and its correlation with treatment response and long-term survival. Meanwhile, SPRED1 hypermethylating cell lines were screened by bisulfite sequencing PCR (BSP) method and the possible relationship between aberrant SPRED1 methylation and leukemogenesis, which was confirmed by 5-aza-2′-deoxycytidine (5-AZA) and overexpressed lentivirus transfection assays in AML cell lines.

## Materials and Methods

### Primary Human Tissue

Bone marrow samples from 75 AML patients (44 men and 31 women) as well as 26 healthy adult samples were obtained at the First Affiliated Hospital of China Medical University in China between February 2015 and September 2017. The median age of patients was 51.5 years (range: 17 to 69 years). The diagnosis and classification were made according to the French-American-British (FAB) Cooperative Group criteria and WHO classification (15). APL, which is the M3 subtype of FAB characterizing those with t(15;17) or PML/RARA fusion gene, was excluded from this study.

The follow-up of 71 patients was done until December 2019. The treatment regimens and response assessment followed the Chinese expert consensus on the treatment of AML (2011). A total of 13 patients received allo-hematopoietic stem cell transplantation (allo-HSCT) in the complete remission (CR) phase. CR was evaluated after the first therapy. This study was approved by the Ethics Committee of the First Affiliated Hospital of China Medical University (#AF-SOP-07-1.0-01).

### Cell Culture

Human-AML cell lines HL-60, THP-1, and OCI-AML2 were obtained from the American Type Culture Collection (ATCC, Manassas, VA, USA) and cultured in Roswell Park Memorial Institute (RPMI)-1640 (GIBCO, Grand Island, NY, USA) medium supplemented with 10% (v/v) fetal bovine serum (HyClone, Logan, UT, USA) at 37°C and 5% CO_2_ in a humidified incubator.

### Karyotype Analysis

Bone marrow cells were extracted in all cases, and chromosome specimens were prepared by direct method and/or short-term culture method. The G-banding technique was used for karyotype analysis; at least 20 metaphase cells were analyzed for each case, which were identified and described according to the International System for Human Cytogenetic Nomenclature (ISCN 1995).

### High-Throughput Next-Generation Sequencing-Based Assay

Next-generation sequencing (NGS) was performed on bone marrow samples with a Genoptix panel by Yuanqi Biomedical Technology Co. Ltd. (Shanghai, China). The panel covers hot spots of 21 AML associated genes including ASXL1, CEBPA, DNMT3A, FLT3, GATA2, IDH1, IDH2, KIT, KRAS, MLL, NPM1, NRAS, PHF6, RUNX1, TET2, TP53, WT1, SF3B1, SRSF2, U2AF1, and ETV6.

### Quantitative High-Resolution Mass Spectrometry-Based Approach Analysis and Bisulfite Sequencing PCR Analysis of Methylation Status

DNA was extracted from the bone marrow of AML patients using a QIAamp DNA Mini Kit (Qiagen, Hilden, Germany). The quantification of DNA methylation was determined using the quantitative high-resolution mass spectrometry-based approach (MassARRAY) platform (Sequenom, CA, USA) by BGI Tech Solutions Co. (Shanghai, China). As a prediction, 12 CpG units were located at the SPRED1 promoter region starting from 310 to 723 (414 bp), but only ten of them were detectable. The sequences for primers were designed using the Sequenom EpiDesigner software (www.epidesigner.com): forward: 5′-AGGATAATGTTGTTGTTGAGGTAGG-3′ and reverse: 5′-CTAAATCCCAAATACTCCCAAATTC-3′. The following reactions were performed as in our previous study (14).

The SPRED1 methylation status of AML cell lines HL-60, THP-1, and OCI-AML2 were tested by the BSP analysis. SPRED1 promoter sequences were retrieved from University of California, Santa Cruz (UCSC) and National Center for Biotechnology Information (NCBI) database, in total 3,300 bp, including the first 2,000 bp of transcription initiation site and the first intron partial sequence. The predicted results of Methprimer showed that the promoter region contained 3 CpG islands, which were longer than 200 bp, and named as Island1, Island2, and Island3 separately from left to right. Primers for the three major CpG islands on the promoter of SPRED1 are listed in **Supplementary Material 1**. BSP was conducted using TaKaRa Taq™ Hot Start Version kit (Tokyo, Japan). BSP conditions for SPRED1 methylation density detection were 10 s at 98°C, 40 cycles for 10 s at 98°C, 30 s at 58°C, 30 s at 72°C, and followed by a final 7 min step at 72°C. Ten independent clones with three pairs of nest primers in each cell line were sequenced (Wanleibio, Shenyang, China).

### RNA Extraction and Quantitative Reverse Transcription PCR

RNA was extracted from cells of bone marrow samples and cell lines using the TRIpure (BioTeke, Wuxi, China) and reverse transcribed using a Super M-MLV Reverse Transcription Reagent Kit (BioTeke) according to the manufacturers’ protocol. Quantitative reverse transcription PCR (RT-PCR) was performed using 2× Power Taq PCR MasterMix (BioTeke) and SYBR Green (Solarbio, Beijing, China) as a double-stranded DNA-specific dye. Specific primer sequences of SPRED1 designed by Sangon Biotech (Shanghai, China) were as follows: forward: 5′-TTTTCTGATCCCTGTTCGTG-3′ and reverse: 5′-TCCAGCAGCTTTATGTTTCC-3′. The following steps of RT-PCR followed protocols in our laboratory. The relative level of SPRED1 was analyzed using the Exicycler™ 96 System (BIONEER, Daejeon, Korea) and calculated using the 2^−ΔΔCt^ method.

### Western Blotting

Cell lines were washed twice with phosphate-buffered saline (PBS) before being lysed with radio-immunoprecipitation assay buffer (Wanleibio). Protein concentrations were determined using the bicinchoninic acid protein assay kit (Wanleibio) according to the manufacturer’s protocol. Equal amounts of protein lysate (30 μg) were separated by 10% sodium dodecyl sulfate–polyacrylamide gel electrophoresis (SDS-PAGE) and transferred onto polyvinylidene difluoride membranes (Millipore, Billerica, MA, USA). After being blocked with 5% skim milk, the membranes were incubated with primary antibodies (1:1,000) against SPRED1 (Abcam, Cambridge, UK, no. 77079), (1:400) against p-ERK (Wanleibio, WLP1512), (1:400) against ERK (Wanleibio, WL01864), (1:500) against cleaved-caspase-3 antibody (Wanleibio, WL01992), (1:500) against p-P53 (Wanleibio, WL03214), (1:1,000) against Bax (Wanleibio, WL01637), (1:1,000) against Bcl2 (Wanleibio, WL01556), and β-actin (Wanleibio, WL01845) overnight at 4°C. The same membrane was washed with Tris-buffered saline with Tween 20 and reblotted with peroxidase-conjugated goat anti-rabbit (1:5,000 Wanleibio, WLA023) for 45 min at 37°C. For quantitative analysis, the bands were selected and quantified using Gel-Pro-Analyzer Software, and the data were transformed and normalized to β-actin.

### Development of an Overexpressed SPRED1 Cell Line

The siRNA-targeting region (GCAGATGACTTACAAGCAA) of the human SPRED1 gene (NM_152594.2, CDS 1,335 bp) was designed, synthesized, and ligated with the Tet-pLKO-puro vector (Addgene). First, we observed the growth state of the THP-1 cells and made sure that the exact volume of the venom was absorbed (the ratio of virus number to THP-1 cell number was 100:1), which was added to the prepared medium. With the culture medium removed from the cells, the calculated virus venom plus medium was added to the target cells. Plates were cultured under the following conditions, shaking for a constant mixture of the media while at 37°C and 5% CO_2_ for 48 h. Afterward, we selected the detection of related indicators.

The Tet-pLKO-puro vector (Addgene) was used as a non-silencing control or for carrying SPRED1 target shRNA. This is the sequence used for SPRED1 shRNA: GCAGATGACTTACAAGCAA. For SPRED1 expression, the vector was constructed by cloning human SPRED1 cDNA (NM_152594.2) into pLV-EF1a-IRES-NEO vector (Addgene) according to the standard procedure. Lentiviral packaging and transductions were carried out using standard procedure and according to the manufacturer’s instructions. Cells transfected with a virus carrying an empty vector were used as a negative control (control group), and untreated cells were used as a blank control (blank group).

### MTT Assay

*Blank group*: THP-1 was cultured for 48 h. *5-AZA treatment*: THP-1 was treated with 5 μM of 5-AZA for 48 h. *Control group*: THP-1 was transfected with an empty vector for 48 h. *Lentivirus transfection* (SPRED1 OE): THP-1 was transfected with SPRED1 overexpressed lentivirus for 48 h. Each blank cell (8 × 10^3^ cells/well) measuring 100 μl was seeded separately in 96-well plates, which had been pre-incubated for 48 h at 37°C in an atmosphere of CO_2_. A total of 0.5 mg/ml of MTT solution was added to each well and incubated for 4 h. The absorbance was measured at a wavelength of 570 nm using a microplate reader (ELX-800, BIOTEK, Winooski, VT, USA).

### Differentiation and Apoptosis Assay by Flow Cytometry

*Blank group*: THP-1 was cultured for 48 h. *5-AZA treatment*: THP-1 was treated with 5 μM of 5-AZA for 48 h. *Control group*: THP-1 was transfected with empty vectors for 48 h. *Lentivirus transfection* (SPRED1 OE): THP-1 was transfected with SPRED1 overexpressed lentivirus for 48 h. The differentiation and apoptosis of each group of cells were assessed by flow cytometry using CD11b (eBioscience, San Diego, CA, USA, 85-11-0118-41), CD14 (eBioscience, 11-0149-41), and the Annexin V/Propidium iodide-fluorescein isothiocyanate Apoptosis Detection kit (Wanleibio, WLA001b), according to the manufacturers’ protocol. Cells treated with PBS were used as negative controls.

### Statistical Analysis

Statistical analysis was performed using IBM SPSS version 23 statistics software. The Mann–Whitney test was used to evaluate the significance of any differences between the patients and controls. A t-test was used to compare the difference between the paired subgroup and cell line data; relevance was determined by Spearman’s correlation analysis. The differences with *p* < 0.05 were considered statistically significant. Overall survival (OS) was calculated from diagnosis to time of death. Relapse-free survival (RFS) was estimated from the date of the CR/CR with incomplete platelet recovery (CRp) to the date of relapse, death, or last contact to patients alive in continuous CR/CRp. The Kaplan–Meier method was used to perform the survival curve, and the log-rank test was used to test statistical significance.`

## Results

### Higher Methylation Level and Lower mRNA Level of SPRED1 in Acute Myeloid Leukemia Patients Compared to the Control Group

**Figure 1:**
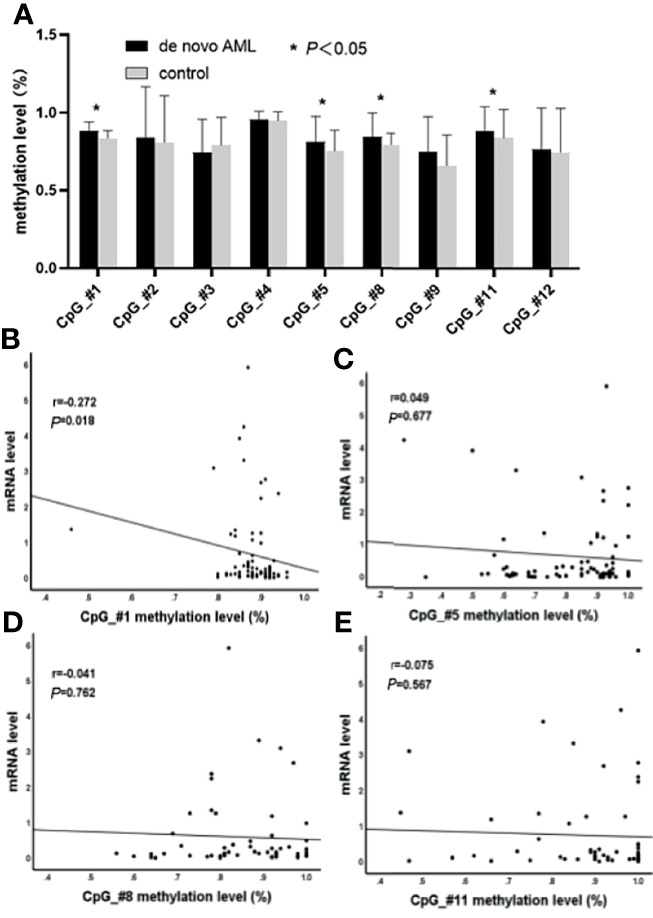
Comparison of nine CpG units (CpG_#1–5, 8–9, and 11–12) in acute myeloid leukemia (AML) to the control group were evaluated. **(A)** The methylation levels are significantly elevated in AML patients at units CpG_#1, CpG_#5, CpG_#8, and CpG_#11 (*p < 0.05). **(B)** Correlation between methylation and mRNA levels of CpG_#1 in AML (*p* < 0.05). **(C)** Correlation between methylation and mRNA levels of CpG_#5 in AML (*p* > 0.05). **(D)** Correlation between methylation and mRNA levels of CpG_#8 in AML (*p* > 0.05). **(E)** Correlation between methylation and mRNA levels of CpG_#11 in AML (*p* > 0.05).

The DNA methylation levels of ten CpG units (CpG_#1–5, 7–9, and 11–12) were determined in a total of 75 AML (non-APL) patients and 26 healthy controls using the MassARRAY platform from Sequenom. All samples contained more than 70% of the data points, and all except one CpG (CpG_#7) unit produced data from more than 70% of samples. All in all, nine CpG units (CpG_#1–5, 8–9, and 11–12) were able to be evaluated. Compared to those of the control group, the methylation levels were significantly elevated in AML patients at units of CpG_#1 (87.93% ± 0.71% *vs*. 83.58% ± 0.96%, *p* = 0.000), CpG_#5 (81.25% ± 1.88% *vs*. 75.31% ± 2.59%, *p* = 0.032), CpG_#8 (84.29% ± 2.03% *vs*. 79.33% ± 1.50%, *p* = 0.013), and CpG_#11 (88.02% ± 1.96% *vs*. 83.67% ± 4.02%, *p* = 0.029) (**Figure 1A**). Decreased expression of SPRED1 mRNA was confirmed in the 75 AML patients compared with the control group (0.660 ± 0.132 *vs*. 19.274 ± 13.599, *p* = 0.000, **Supplementary Material 2**). The methylation level of CpG_#1 and the SPRED1 mRNA level were negatively correlated (*r* = –0.272, *p = 0.018, **Figure 1B**). However, no significant correlation was observed between the methylation level and SPRED1 mRNA expression at CpG_#5 (*r* = 0.049, *p* = 0.677), CpG_#8 (*r* = –0.041, *p* = 0.762), and CpG_#11 (*r* = –0.075, *p* = 0.567) (**Figures 1C–E**).

### Relationship of Methylation Level of SPRED1 and Clinical Parameters in Acute Myeloid Leukemia

The correlation between the hypermethylation level of SPRED1 and the clinical parameters of AML was investigated in **Table 1**. Among the 75 patients, the methylation level of CpG_#1 was higher in younger (<60) than in older patients (≥60) (*p* = 0.022). The methylation level of CpG_#8 was higher in female patients than in male patients (*p* = 0.025). There was no significant difference between the methylation level and the peripheral blood counts [white blood cells (WBC), hemoglobin (Hb), and platelets (PLT)], bone marrow blasts ratio, FAB subtypes, and karyotypic abnormalities.

**Table 1:** SPRED1 methylation levels in AMLs with different clinical parameters.

Parameters	Number (%)	CpG_#1 level (%) average ± SD	Number (%)	CpG_#5 level (%) average ± SD	Number (%)	CpG_#8 level (%) average ± SD	Number (%)	CpG_#11 level (%) average ± SD
**Age (years)**								
≥ 60	11 (14.7%)	82.55 ± 3.843*	11 (14.7%)	80.45 ± 4.646	9 (15.52%)	81.56 ± 9.770	7 (11.48%)	78.29 ± 8.815
<60	64 (85.3%)	88.86 ± 0.443*	64 (85.3%)	81.39 ± 2.070	49 (84.48%)	84.80 ± 1.686	54 (88.52%)	89.28 ± 1.868
**Gender**								
Male	44 (58.67%)	88.14 ± 0.548	44 (58.67%)	80.11 ± 2.535	35 (60.34%)	82.49 ± 2.038*	37 (60.66%)	89.32 ± 2.168
Female	31 (41.33%)	87.65 ± 1.545	31 (58.67%)	82.87 ± 2.812	23 (39.66%)	87.04 ± 4.076*	24 (39.34%)	86.00 ± 3.705
**Peripheral blood count**								
WBC ≥10 × 10⁹/L	46 (61.3%)	87.63 ± 1.058	46 (61.3%)	78.98 ± 2.509	35 (60.34%)	84.29 ± 2.934	39 (63.93%)	89.44 ± 2.328
WBC < 10 × 10⁹/L	29 (38.7%)	88.41 ± 0.755	29 (38.7%)	84.86 ± 2.719	23 (39.66%)	84.30 ± 2.589	22 (36.07%)	85.50 ± 3.527
Hb ≥ 80 g/L	42 (56%)	87.62 ± 0.590	42 (56%)	79.14 ± 2.440	31 (53.45%)	85.74 ± 1.920	30 (49.18%)	88.47 ± 2.901
Hb < 80 g/L	33 (44%)	88.33 ± 1.437	33 (44%)	83.94 ± 2.916	27 (46.55%)	82.63 ± 3.786	31 (50.82%)	87.58 ± 2.677
PLT ≥ 50 × 10^9^/L	29 (38.7%)	88.69 ± 0.647	29 (38.7%)	83.52 ± 2.495	27 (46.55%)	83.59 ± 2.361	24 (39.34%)	91.33 ± 2.601
PLT < 50 × 10^9^/L	46 (61.3%)	87.46 ± 1.082	46 (61.3%)	79.83 ± 2.634	31 (53.45%)	84.90 ± 0.323	37 (60.66%)	85.86 ± 2.717
**Bone marrow** (**n lost = 6**)								
Blast% ≥ 65	33 (47.83%)	88.21 ± 1.426	33 (47.83%)	82.15 ± 2.468	26 (47.27%)	84.15 ± 3.781	29 (51.79%)	88.86 ± 2.918
Blast% < 65	36 (52.17%)	87.44 ± 0.682	36 (52.17%)	81.94 ± 3.011	29 (52.73%)	84.03 ± 2.197	27 (48.21%)	86.44 ± 3.066
**FAB classification**								
M0	1 (1.33%)							
M1	0							
M2	26 (34.67%)	88.96 ± 0.760	26 (34.67%)	83.62 ± 3.120	20 (34.48%)	84.95 ± 2.641	20 (32.79%)	90.55 ± 2.914
M3 (exclude)	0							
M4	2 (2.67%)							
M5	44 (58.67%)	87.48 ± 1.104	44 (58.67%)	80.30 ± 2.443	33 (56.90%)	84.88 ± 3.125	37 (60.66%)	87.32 ± 2.653
M6	2 (2.67%)							
M7	0							
**No. of karyotypic abnormalities (n lost = 21)**								
Normal	31 (57.41%)	87.87 ± 0.647	31 (57.41%)	78.06 ± 2.972	22 (55%)	86.55 ± 2.469	20 (48.78%)	84.15 ± 3.711
t(8;21)	8 (14.81%)	83.00 ± 5.375	8 (14.81%)	89.25 ± 2.902	7 (17.5%)	78.43 ± 11.946	7 (17.07%)	76.00 ± 8.232
t(16;16)/inv(16;16)	5 (9.26%)	87.00 ± 1.265	5 (9.26%)	65.00 ± 13.506	2 (5%)	87.50 ± 9.500	4 (9.76%)	91.50 ± 4.787
complex	2 (3.70%)	87.50 ± 3.500	2 (3.70%)	84.00 ± 6.000	2 (5%)	85.50 ± 7.500	2 (4.88%)	85.50 ± 8.500
others	8 (14.81%)	86.88 ± 1.807	8 (14.81%)	77.63 ± 4.105	7 (17.5%)	80.57 ± 5.498	8 (19.51%)	91.25 ± 6.472

AML, acute myeloid leukemia; WBC, white blood cells; Hb, hemoglobin; PLT, platelets.

The methylation level of CpG_#1 was higher in younger (<60) than in older patients (≥60) (*p = 0.022). The methylation level of CpG_#8 was higher in female patients than in male patients (*p = 0.025).

A total of 144 hot spot mutations, involving 22 genes, were first identified by NGS and afterward divided into six subgroups, based on their underlying biological function (**Supplementary Material 3**). The correlation between methylation level and the common mutations (frequency ≥ 3), involving FLT3-ITD, NPM1, TET2, and CEBPA, was analyzed in 31 AML cases with a normal karyotype (NK-AML). A significantly increased methylation level of CpG_#8 was detected in FLT3-ITD and NPM1 mutations (**Supplementary Material 4**).

### Association of SPRED1 Methylation Levels With Treatment Response and Survival

**Figure 2:**
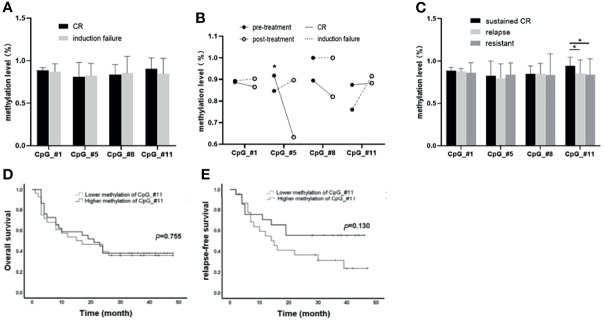
Association of SPRED1 methylation levels with treatment response and survival. **(A)** Differences in methylation levels of the four CpGs between complete remission (CR) and induction failure (*p* > 0.05). **(B)** Differences on paired methylation levels of the four CpGs between the pre- and post-induction treatment from 4 CR and 3 induction failure patients. * The methylation of post-treatment was significantly lower than that of pre-treatment at CpG_#8 in 4 CR patients (91.75%±5.91% vs. 63.25%±3.86%, *p = 0.002). **(C)** Differences in methylation levels of the four CpGs among sustained CR, relapsed, and never CR (resistant). *CpG_#11 showed a significantly increased methylation level (94.00%±2.417%) in sustained CR compared to relapsed patients (85.12% ± 3.236%, *p = 0.015) and no CR (83.92%±5.158%, *p = 0.041). **(D, E)** Differences in overall survival (OS) and relapse-free survival (RFS) between hypermethylation and hypomethylation subgroups at CpGs_#11 (*p* > 0.05).

All 75 patients accepted induction treatments: 50 of them achieved CR after the first induction, while 25 were considered induction failure. Between the two groups, there was no significant difference in methylation levels in the four CpGs (#1: 88.44% ± 0.50% *vs*. 86.92% ± 1.89%, *p* = 0.968; #5: 80.82% ± 2.43% *vs*. 82.12% ± 2.95%, *p* = 0.839; #8: 83.65% ± 2.01% *vs*. 85.21% ± 4.06%, *p* = 0.176; #11: 90.22% ± 2.13% *vs*. 84.63% ± 3.69%, *p* = 0.320; **Figure 2A**). However, paired methylation levels (pre- and post-induction treatment) of the four CpGs from seven patients (CR n = 4, failure n = 3) showed that the methylation of post-treatment was lower than that of pre-treatment at CpG_#1, CpG_#5, and CpG_#8, except at CpG_#11 in CR patients, while being higher than that of pre-treatment at CpG_#1, CpG_#5, and CpG_#11 in induction failure (**Figure 2B**).

The median time of follow-up of the 71 patients who accepted consolidation treatment was 17 months (2 to 24 months). Out of the 71 patients, 29 patients were in sustained CR, 28 relapsed, and 14 were considered resistant because of no CR. It is worth noting that CpG_#11 showed a significantly increased methylation level (94.00% ± 2.417%) in sustained CR compared to relapsed patients (85.12% ± 3.236%, *p = 0.015) and no CR (83.92% ± 5.158%, **p = 0.041) (**Figure 2C**). However, there was no statistical difference for OS and RFS between hypermethylation and hypomethylation subgroups at either CpGs_#11 (**Figures 2D, E**) or the other three CpGs (**Supplementary Material 5**).

### THP-1, the Acute Myeloid Leukemia Cell Line With the Highest Methylation Level and the Lowest mRNA and Protein Expression of SPRED1

**Figure 3:**
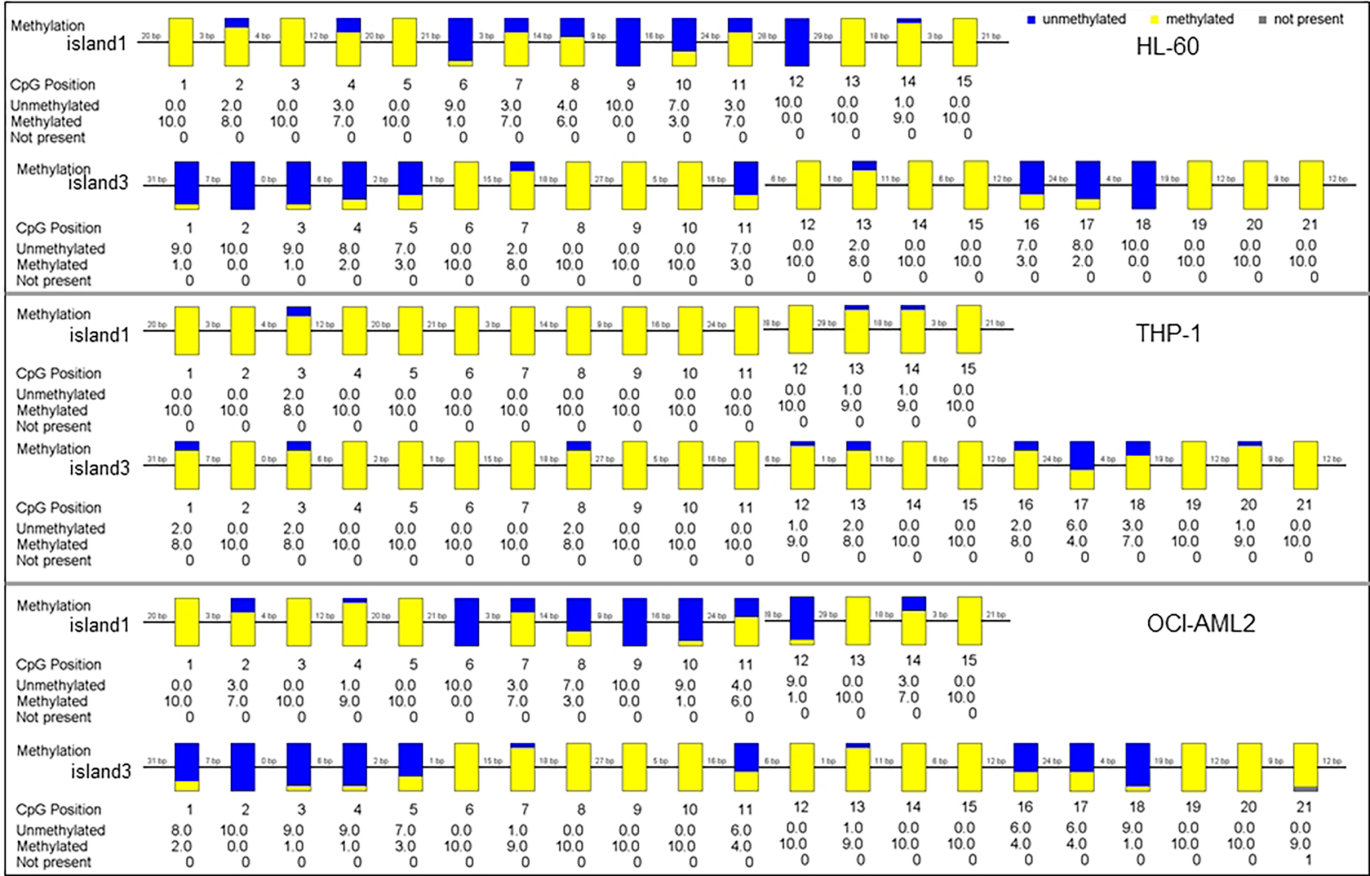
The methylation status of SPRED1 CpG islands in three acute myeloid leukemia (AML) cell lines.

The methylation status of three CpG islands was determined in AML cell lines by BSP assay. Methylation values in Island1 and Island3 were evaluated in ten clones of each cell line, including a total of 36 CpG sites, 15 within Island1 (275 bp) and 21 within Island3 (286 bp) (**Figure 3**).

**Figure 4:**
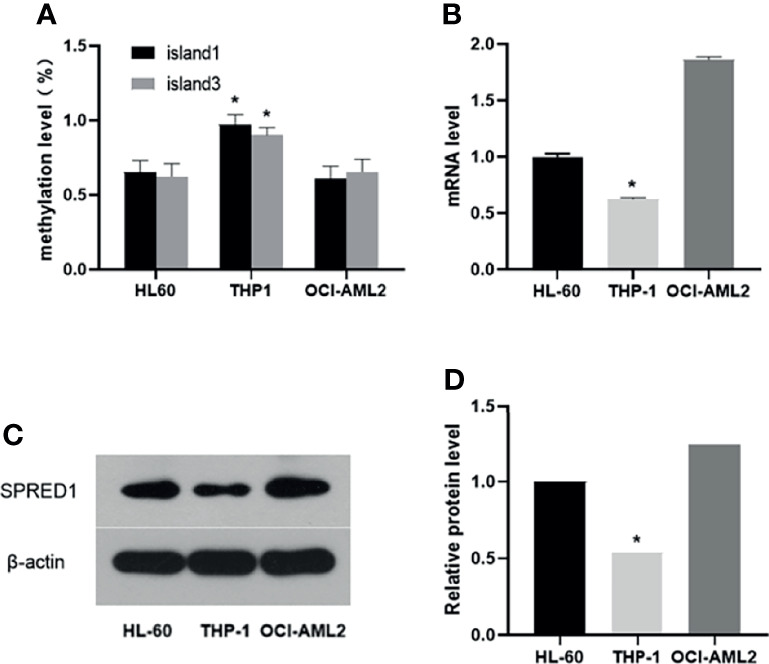
The methylation, mRNA, and protein levels of SPRED1 in three acute myeloid leukemia (AML) cell lines. **(A)** The methylation levels of Island1 and Island3 in 3 AML cell lines (*p < 0.05). **(B)** The mRNA levels of 3 AML cell lines (*p < 0.05). **(C)** The protein levels of 3 AML cell lines by Western blotting. **(D)** Relative protein quantification of Western blotting by densitometric analysis (*p < 0.05).

In HL-60 cell line, 98 of 150 cases showed a methylated C at Island1 (average level 0.653 ± 0.076), and 131 of 210 cases were methylated at Island3 (average level 0.623 ± 0.086). In THP-1, 146 cases carried methylated C at Island1 (average level 0.973 ± 0.064) and 189 cases methylated at Island3 (average level is 0.900 ± 0.052). In OCI-AML2, 91 cases presented with methylated C at Island1 (average level is 0.606 ± 0.086) and 137 cases methylated at Island3 (average level is 0.655 ± 0.083). THP-1 cell showed the highest methylation levels in both Island1 and Island3, compared with HL-60 (Island1: *p* = 0.000, Island3: *p* = 0.000) and OCI-AML2 (Island1: *p* = 0.000, Island3: *p* = 0.000) (**Figure 4A**). Correspondingly, the lowest mRNA and protein expressions were detected in THP-1 (0.62 ± 0.01, *p* = 0.000; 0.54, *p* = 0.000) compared to the other two cell lines (HL-60: 1.00 ± 0.02; 1.00) (OCI-AML2: 1.86 ± 0.03; 1.24) (**Figures 4B–D**).

### 5-AZA Treatment Upregulates SPRED1 Expression and Inactivates p-ERK

**Figure 5:**
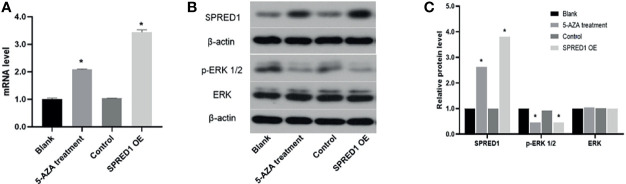
SPRED1 mRNA level, SPRED1 protein level, and p-ERK/ERK protein levels of THP-1 after treatment with 5 μM of 5-AZA and lentivirus transfection. **(A)** SPRED1 mRNA level of THP-1 after treatment with 5 μM of 5-AZA and lentivirus transfection (*p < 0.05). **(B)** SPRED1 protein level and p-ERK/ERK protein level of THP-1 after treatment with 5 μM 5-AZA and lentivirus transfection by Western blotting. **(C)** Relative protein quantification of Western blotting by densitometric analysis (*p < 0.05).

The impact of hypomethylation on SPRED1 expression and its downstream signaling pathway was further investigated in THP-1 by a 5-AZA treatment assay. As shown in **Figure 5A**, the SPRED1 mRNA expression after treatment with 5-AZA for 48 h (2.09 ± 0.03) was significantly higher than the blank (1.00 ± 0.04, *p* = 0.000), as well as the level of overexpressed lentivirus transfection (SPRED1 OE) (3.44 ± 0.08) was significantly higher than the control (1.04 ± 0.01, *p* = 0.000). Moreover, it showed that the protein of SPRED1 was also overexpressed after 5-AZA treatment (relative level: 2.63, *p* = 0.000) and lentivirus transfection (relative level: 3.81, *p* = 0.000) in contrast with the blank group (relative level: 1.00), and with the control (relative level: 1.00) (**Figures 5B, C**).

Expression of p-ERK1/2, which is the downstream protein of SPRED1, significantly decreased in 5-AZA treatment cells (relative level: 0.45, *p* = 0.000) and lentivirus transfection cells (relative level: 0.45, *p* = 0.000), compared to the blank (relative level: 1.00), and the control as well (relative level: 0.92). There was no difference in ERK protein level among the cells of 5-AZA treatment (relative level: 1.04), lentivirus transfection (relative level: 1.00), blank (relative level: 1.00), and control (relative level: 1.02) (**Figures 5B, C**).

### 5-AZA Treatment Inhibits Cell Proliferation and Promotes Cell Differentiation

**Figure 6:**
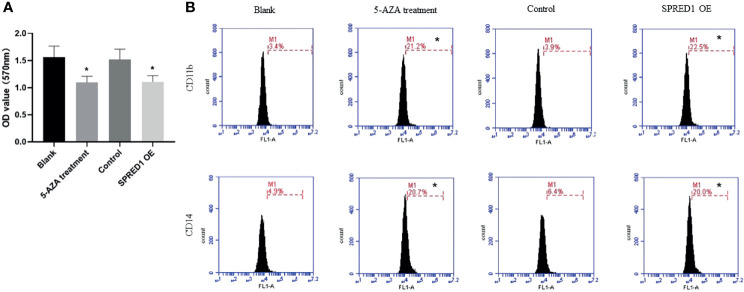
Cell proliferation and differentiation of THP-1 after treatment with 5 μM of 5-AZA and lentivirus transfection. **(A)** The proliferative capacity of THP-1 after treatment with 5 μM of 5-AZA and lentivirus transfection by MTT assay (*p < 0.05). **(B)** CD11b and CD14 expressions of THP-1 after treatment with 5 μM of 5-AZA and lentivirus transfection by flow cytometry (*p < 0.05).

The proliferative capacity of THP-1 was evaluated by MTT assay as shown in **Figure 6A**. 5-AZA treatment cells presented with significantly decreased cell proliferation (1.097 ± 0.111) compared with the blank (1.563 ± 0.203, *p* = 0.004), same as the lentivirus transfection cells (1.109 ± 0.112) compared with the control (1.519 ± 0.190, *p* = 0.003), respectively.

In addition, the expression of differentiation markers CD11b and CD14 were significantly elevated in THP-1 with 5-AZA treatment (CD11b: 21.2% ± 0.072%; CD14: 20.7% ± 0.042%) compared to the blank (CD11b: 3.4% ± 0.445%; CD14: 4.9% ± 0.945%, *p* = 0.000), as well as the lentivirus transfection cells (CD11b: 22.5% ± 0.057%; CD14: 20.0% ± 0.081%) compared with the control (CD11b: 3.9% ± 0.040%, CD14: 6.4% ± 0.350%, *p* = 0.000) (**Figure 6B**).

### 5-AZA Treatment Promotes Cell Apoptosis

**Figure 7:**
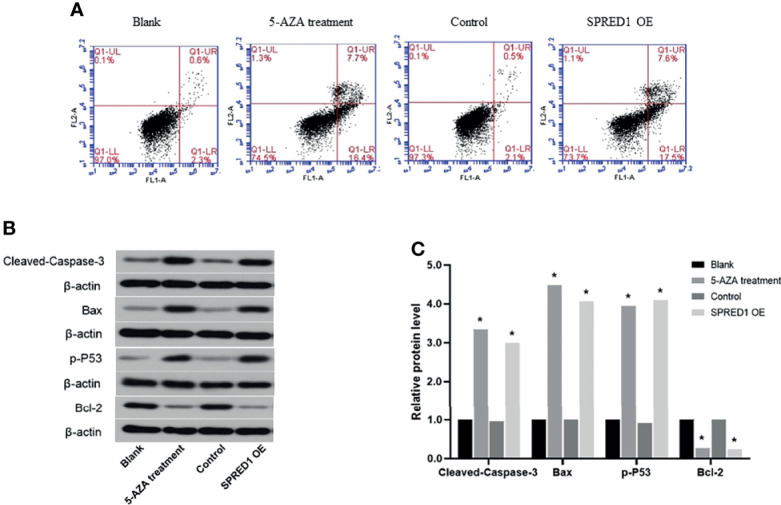
Activation of apoptosis following the treatment with 5 μM of 5-AZA and lentivirus transfection. **(A)** Cell apoptosis ratios of THP-1 after treatment with 5 μM of 5-AZA and lentivirus transfection by flow cytometry (*p < 0.05). **(B)** Biomarkers of apoptosis-related proteins, including Cleaved Caspase-3, Bax, p-P53, and Bcl-2 of THP-1, after treatment 5 μM of 5-AZA and lentivirus transfection by Western blotting. **(C)** Relative protein quantification of Western blotting by densitometric analysis (*p < 0.05).

Apoptosis ratios were determined by flow cytometry in 5-AZA treatment cells, which were significantly lower (7.7% ± 0.003%) than those in the blank (0.6% ± 0.010%, *p* = 0.000) and lentivirus transfection cells (7. 6% ± 0.090%) and also much lower than those in the control (0.5% ± 0.050%, *p* = 0.000) (**Figure 7A**).

Biomarkers of apoptosis-related proteins, including Cleaved Caspase-3, Bax, p-P53, and Bcl-2, were further evaluated (**Figures 7B, C**). The relative protein levels of Cleaved Caspase-3, Bax, p-P53, and Bcl-2 in 5-AZA treatment cells compared to the blank group were 3.34, 4.49, 3.94, and 0.27, respectively (*p* = 0.000). Similar to that, the relative protein levels of Cleaved Caspase-3, Bax, p-P53, and Bcl-2 in lentivirus transfection cells compared to the control group were 3.00, 4.07, 4.10, and 0.24, respectively (*p* = 0.000). There was no significant difference between the blank and the control.

## Discussion

Nowadays, the treatment strategies for AML are chosen based on age and prognostic stratification, which is determined by cytogenetic and molecular alterations (16–18). Thanks to recent technological advancement in the field of genome research, studying genomic changes and epigenetic modifications in AML cell lines, such as abnormal DNA methylation, has gained more and more interest (19–21), while using the demethylating agents (HMAs) is definitely effective, especially for patients who are implicated in abnormalities of epigenetic regulation (22, 23). However, HMAs yield low response rates (10%–50%, including hematologic improvement) and are not curative, with a median OS of less than 1 year. Thus, there is a critical need to develop targets capable of inducing a deep and persistent clinical response (24, 25).

In our prior studies, we found that the expression of SPRED1 was significantly reduced, which was closely related to the hypermethylation of SPRED1 in non-APL patients, and the patients who obtained CR might be with decreasing methylation level of SPRED1 (14). Also, our results indicated that the methylation status of SPRED1 might be associated with treatment response of AML and implicated a treatment failure to traditional chemotherapy. However, the clinical prognostic significance of SPRED1 remains to be further proven in view of the limitation of the clinical sample size. In this study, the prognostic impact of abnormal SPRED1 methylation on AML was assessed in a large cohort of patients. It seems that the methylation level of SPRED1 CpG is a marker of the treatment response to induction therapy (CR or not) and remission duration (sustained CR), but not a predictor of long-term survival. The patients with lower methylation of CpGs after induction treatment than before could get CR, while neither RFS nor OS was dependent on SPRED1 hypermethylation status. This suggests that the hypermethylation of SPRED1 might be implicated in the resistance to chemotherapy since all patients in this study accepted combined chemotherapy. Whether the patients with abnormal methylating status might benefit from HMAs is worth further investigation.

Our previous pilot study has shown that SPRED1 hypermethylation was related to some common AML gene mutations, such as DNMT3A and TET2 (14), which were response indicators for demethylation agents (26, 27). However, in this study, hypermethylation of SPRED1 is more frequently presented in patients with an FLT3-ITD or NPM1 mutation, but not gene mutations involving DNA methylation, indicating that hypermethylation of SPRED1 might mediate resistance to drug by a more complex mechanism. Therefore, we determined the demethylation influence on cell lines with bisulfite modification, followed by PCR and sequencing, which is the most effective method for the high-resolution methylation mapping of genomes (28). As a DNA methyltransferase inhibitor, 5-AZA is a clinically used epigenetic agent that induces promoter demethylation and gene re-expression AML (29, 30). It confirmed that the methylation level of SPERD1 is negatively correlated with mRNA or protein level, and after treatment of 5-AZA, the mRNA and protein levels of SPRED1 increased significantly. It might be due to the CpGs considerably expanding; it elicited abnormal DNA methylations, which were responsible for SPRED1 transcriptional silencing. Meanwhile, SPRED1 overexpressed through the way of lentivirus transfection, the proliferation activity of AML cells declined, and the proportion of differentiation into mature cells and apoptosis increased. We suggested that in AML, the methylation of SPRED1 might be associated with the malignant transformation, abnormal differentiation, and maturation of hematopoietic stem cells. SPRED1 overexpression also significantly inhibits the expression of p-ERK, which proves that SPRED1 may be another target to reverse AML progression. Similar to overexpression of SPRED1, demethylation could upregulate the expression of SPRED1, thus inhibiting the ERK/MAPK signaling pathway to promote cell apoptosis. Therefore, SPRED1 is a potential target for demethylation therapy in AML.

In conclusion, our study demonstrates that in AMLs, SPRED1 is hypermethylated, and de-hypermethylation of SPRED1 could inhibit the proliferation of AML cells. This also promotes the differentiation and apoptosis of AML cells, and its mechanism is possibly related to the ERK pathway. Meanwhile, knockout and/or transgenic animal models may accurately reflect inter-individual differences among AMLs, by which we may further understand how SPRED1 hypermethylation is relevant to leukemogenesis, including the conjugation of SPRED1 methylation with other molecular markers. At the same time, it will help us understand how SPRED1/ERK crosstalk signaling works, which can probably support a new epigenetic therapy target for AMLs. Meanwhile, whether the combination of demethylating agents and SPRED1 targeting agents can further improve the efficacy of AML therapy needs further exploration in the near future.

## Data Availability Statement

The datasets presented in this study can be found in online repositories. The names of the repository/repositories and accession number(s) can be found below: NCBI with accession: PRJNA807494, (http://www.ncbi.nlm.nih.gov/bioproject/PRJNA807494).

## Ethics Statement

The studies involving human participants were reviewed and approved by the Ethics Committee of the First Affiliated Hospital of China Medical University (#AF-SOP-07-1.0-01). The patients/participants provided their written informed consent to participate in this study.

## Author Contributions

NS wrote the first draft of the manuscript. YW performed the statistical analysis and collected the data. YW and RZ wrote sections of the manuscript. RZ contributed to the conception and design of the study. XL, WX, HP, and HW performed sections of the experiments. WM and RT made statistical analysis. SL, XY, and YL contributed to conception. All authors listed have made a substantial, direct, and intellectual contribution to the work and approved it for publication.

## Funding

This work was supported by the “Shenyang Young and Middle-aged Science and Technology Innovation Talents Support Plan” (Grant number: RC170525), “Natural Science Foundation of Liaoning Province” (Grant number: 2021-MS-190), and “National Natural Science Foundation of China” (Grant number: 81600117).

## Conflict of Interest

The authors declare that the research was conducted in the absence of any commercial or financial relationships that could be construed as a potential conflict of interest.

## Publisher’s Note

All claims expressed in this article are solely those of the authors and do not necessarily represent those of their affiliated organizations, or those of the publisher, the editors and the reviewers. Any product that may be evaluated in this article, or claim that may be made by its manufacturer, is not guaranteed or endorsed by the publisher.
