# Supplementary material for: Methylation of SPRED1: A New Target in Acute Myeloid Leukemia
Source: Front Oncol. 2022 Mar 10;12:854192. doi: 10.3389/fonc.2022.854192 (PMC8960233; doi:10.3389/fonc.2022.854192)
Supplement: Supplementary file 1 [file DataSheet_1.docx]

SI 1. Primers for the three major CpG islands of SPRED1 promoter.

**Island 1: 275bp (1137-1411)**

gtgacagctgtctatccaatcggtccgcctccgttcagagcagttcgtgccacagaagcatctcctgcgctatagttatgcaacctcactcgcagcgggagttatttcctccgttctggctgcgccagattcatgtaacacgcctaccctgggcttcaatggaagtcgttctccattaacaatcactccagctttccgagatgaatgccctgatagcataccccttgcgcagtaagaatccaggcagcgaggcgaggtgttaaaacacacataca

**Island 2: 1071bp (1694-2764)**

cacactgtctttctagcctcttcgttcaagcaacccggatattccaagcagagtaacggggagtaacgcgccacaacaatgcaacctccctagcagctcccctcgcagctcccacccccgccccctttcgcggcgtgccaagagtttggagcgcgcgcacgcacaccgagcacggtgcagggaggatatccctccgcctccctccccctttctccgctaagtggctcgacaacgatttgggaaatgaagggaggagggaactactttcctgaaacttgctatgctgcacatgacttcgagttgcagtGATTCGCCCGGAGGCCGCGGCTTTTAGTCCTACCCACACACCCTCACAGCCTGGGCACCCCGCTCGGCAGCCCCACCCCGCGCCCGCTCTGGCCTCCGGGGAAAGCGGGCGGCCGGGAGAAGTCGCGAGGGACTACTTCGCCTCCTTCTCTCGCGGGCGCCCCCGGTTCGGGCAGCGGCTGCGGCGGCGGAAGGAGCGGGCGGCGTGAGCGCTTCCGCCGCCCCCCTCGCGGCTCCCCTCTCTCGGGCGTGAGGAGCTGGCCCGCTGAGCTGCTGGTGGGCACTGGCTGGCGGGGAACCTGCAGCCGCTCTTGTCCTCTCCCGCCGCTTCTCGGGATCCTGCCCCCCGGACCCGGGCCCGCCCGCTCTTCTCCGGCCGGGTGGGAACGATCGGTTTGCCTGCCCCTCGCCTCACACGCTCCCCGCCGCCCCCTCCACCCCCCTGAGGAGGAGTCCCGGCTGGAGCGTGTCTGGAGGAATCCGCCGCCGCGGGGCCCCTAGCCCTGCTCTGCCGGCGCCTGAGGACCGACCCAGACGCCGCCGGGGGTTGAGGGGCAGCCGGCGGCGAGGCGGGAGATGGTGGGGTGGGCTCCGGCAGGACCGCGCCGCCGCTGCCCGGAGCCTGGGCTCGGCACCCCCCCGCCGGCCCCCACCGAGCGGAGCTCTGCTTCCTCCTCGCCGCATCCCTGAGGGAAGCGCCGCCTCTGCTCCCGGGTTCTCCGCCCGCCGACTCCTTCCTGGCCGGACCCCGCTGCGCTCCACCCC

**Island 3: 286bp (2957-3242)**

TGAGGCATCCACCATGGTGAGGCCCCTGTGCCGCTGCCCCCGCGCCCCCCCGGCCGCCGCTGCCTCCTGCCCCTCGGTGCTGCTGTTGCTCCCCCGCCTGCTGTTGCTCCTCCATCTCCAGATCGGATCACGGTGAGGGAAAGATGAGCGAGGAGACGGCGACTTCTGACAACGAgtaagcgcctcattgatctcgattgctaatccccctccccctatccgccctcggctctcccccagacccatccgaaacttgggtgccggaaagcttgcgaccctggagagt

SI 2. The expression of SPRED1 mRNA in 75 AML patients.


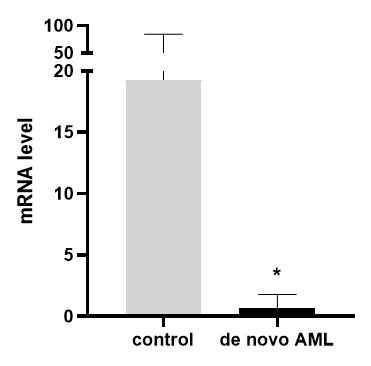


SI 3. The subgroups of gene mutations corresponding with the implicated cellular mode of action


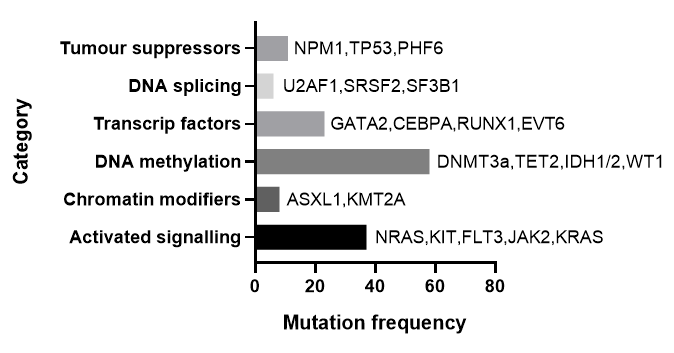


| SI 4. Comparison of gene mutations by SPRED1 methylation levels in NK-AMLs | | | | | | | | | | | | |  |
| --- | --- | --- | --- | --- | --- | --- | --- | --- | --- | --- | --- | --- | --- |
| **Gene Mutations** | | **Number (%)** | **CpG_#1 level (%) average±SD** | ***P*** | **Number (%)** | **CpG_#5 level (%) average±SD** | ***P*** | **Number (%)** | **CpG_#8 level (%) average±SD** | ***P*** | **Number (%)** | **CpG_#11 level (%) average±SD** | ***P*** |
| **FLT3-ITD** | +^a^ | 13（41.94%） | 88.15±1.19 | 0.798 | 13（41.94%） | 80.77±3.97 | 0.567 | 10（45.45%） | 92.00±2.86 | 0.025 | 9（45.00%） | 81.56±6.15 | 0.603 |
|  | -^b^ | 18（58.06%） | 87.67±0.74 |  | 18（58.06%） | 76.11±4.28 |  | 12（54.55%） | 82.00±3.41 |  | 11（55.00%） | 86.27±4.70 |  |
| **NPM1** | + ^a^ | 7（22.58%） | 88.29±2.01 | 0.800 | 7（22.58%） | 83.71±4.17 | 0.317 | 5（22.73%） | 94.20±3.85 | 0.048 | 4（20.00%） | 75.25±11.76 | 0.494 |
|  | - ^b^ | 24（77.42%） | 87.75±0.63 |  | 24（77.42%） | 76.42±3.61 |  | 17（77.27%） | 84.29±2.81 |  | 16（80.00%） | 86.38±3.64 |  |
| **TET2** | + ^a^ | 15（48.39%） | 87.40±1.01 | 0.711 | 15（48.39%） | 77.00±4.91 | 0.892 | 10（45.45%） | 87.80±4.37 | 0.497 | 11（36.67%） | 82.73±4.54 | 0.603 |
|  | - ^b^ | 16（51.61%） | 88.31±0.84 |  | 16（51.61%） | 79.06±3.61 |  | 12（54.55%） | 85.50±2.85 |  | 19（63.33%） | 85.89±6.37 |  |
| **CEBPA**^double/single^ | + ^a^ | 13（41.94%） | 88.00±0.91 | 0.679 | 13（41.94%） | 77.08±5.54 | 0.984 | 10（45.45%） | 83.10±4.00 | 0.203 | 9（45.00%） | 89.33±4.93 | 0.175 |
|  | - ^b^ | 18（58.06%） | 87.78±0.92 |  | 18（58.06%） | 78.78±3.34 |  | 12（54.55%） | 89.42±2.97 |  | 11（55.00%） | 79.91±5.26 |  |

^a^+: mutated/present

^b^-: wildtype/absent

SI 5. The impacts of SPRED1 methylation levels at CpG_#1, 5, 8 on OS and RFS

***P***

***P***

***P***

***P***

***P***

***P***

Fig10. The highest methylation level , the lowest mRNA and protein expression of SPRED1 inTHP-1 cell line.

HL-60

THP-1

OCI-AML2

*
